# Mitochondrial Function in Antarctic Nototheniids with *ND6* Translocation

**DOI:** 10.1371/journal.pone.0031860

**Published:** 2012-02-21

**Authors:** Felix C. Mark, Magnus Lucassen, Anneli Strobel, Esteban Barrera-Oro, Nils Koschnick, Lorenzo Zane, Tomaso Patarnello, Hans O. Pörtner, Chiara Papetti

**Affiliations:** 1 Alfred Wegener Institute for Polar and Marine Research, Bremerhaven, Germany; 2 Instituto Antártico Argentino and CONICET, Buenos Aires, Argentina; 3 Department of Biology, University of Padova, Padova, Italy; 4 Department of Public Health, Comparative Pathology and Veterinary Hygiene, University of Padova, Legnaro, Italy; Université Joseph Fourier, France

## Abstract

Fish of the suborder Notothenioidei have successfully radiated into the Southern Ocean and today comprise the dominant fish sub-order in Antarctic waters in terms of biomass and species abundance. During evolution in the cold and stable Antarctic climate, the Antarctic lineage of notothenioids developed several unique physiological adaptations, which make them extremely vulnerable to the rapid warming of Antarctic waters currently observed. Only recently, a further phenomenon exclusive to notothenioid fish was reported: the translocation of the mitochondrial gene encoding the NADH Dehydrogenase subunit 6 (*ND6*), an indispensable part of complex I in the mitochondrial electron transport system.

This study investigated the potential physiological consequences of *ND6* translocation for the function and thermal sensitivity of the electron transport system in isolated liver mitochondria of the two nototheniid species *Notothenia coriiceps* and *Notothenia rossii*, with special attention to the contributions of complex I (NADH DH) and complex II (Succinate DH) to oxidative phosphorylation. Furthermore, enzymatic activities of NADH∶Cytochrome c Oxidoreductase and Cytochrome C Oxidase were measured in membrane-enriched tissue extracts.

During acute thermal challenge (0–15°C), capacities of mitochondrial respiration and enzymatic function in the liver could only be increased until 9°C. Mitochondrial complex I (NADH Dehydrogenase) was fully functional but displayed a higher thermal sensitivity than the other complexes of the electron transport system, which may specifically result from its unique amino acid composition, revealing a lower degree of stability in notothenioids in general. We interpret the translocation of *ND6* as functionally neutral but the change in amino acid sequence as adaptive and supportive of cold stenothermy in Antarctic nototheniids. From these findings, an enhanced sensitivity to ocean warming can be deduced for Antarctic notothenioid fish.

## Introduction

Antarctic marine life is broadly associated with successful metabolic adaptation to a cold environment, where low temperature and higher oxygen solubility in water are strong selective factors and are paralleled by a specialisation of animals to limited thermal windows [1]. Fish living in this environment are considered highly stenothermal, and their adaptations have been verified at the molecular, cellular, tissue, and organismal hierarchical organisation levels [2], [3], [4], [5]. In this respect, the Antarctic lineage of the perciform suborder Notothenioidei that radiated from a single ancestral benthic fish group within the Southern Ocean displays the most remarkable adaptations, most notably in the gain of antifreeze glycoproteins [6], [7], the loss of haemoglobin and red blood cells in the derived icefish family (Channichthyidae) [8], and the loss of myoglobin in six species within the channichtids [9].

Besides a cold adapted metabolism [10], [11], [12] and concomitant high stenothermy in this group [13], [14], [15], [16], some metabolic plasticity and level of acclimation is possible: warm acclimation can lead to a shift of heat tolerance limits to higher temperatures [17], [18] and involve metabolic compensation [19] at the expense of reduced performance at low temperatures [20]. Similarly, long-term warm acclimation of the Antarctic eelpout *P. brachycephalum* involves metabolic rearrangements [21] and indicates an improvement of hepatic metabolism accompanied by a shift of energy sources [22], [23]. In this context, studies of energy allocation in isolated cells of Antarctic notothenioids have suggested that within a thermal range of about −1°C to 12°C, thermal tolerance limits are defined at whole organism level, e.g. by capacity limitations of the circulatory system rather than by a general failure of cellular energy metabolism [24], [25].

A number of studies have investigated general mitochondrial functionality and capacities in Antarctic fish [26], [27], [28], [29] as key functional traits in thermal acclimatisation and adaptation as they mediate the integration of molecular adaptations into higher functional levels and reflect the energy demand of cells, tissues and organisms under given environmental conditions [30]. Consisting of some 1500 proteins, mitochondria are complex organelles that besides producing ATP through oxidative phosphorylation are involved in a broad array of cellular functions [31], such as calcium storage, apoptosis regulation [32], amino acid and haeme biosynthesis [33], and production of nitric oxide and oxygen radicals. In ectotherms, this complex enzymatic system is strongly influenced by temperature [34].

Studies of mitochondrial capacities in Antarctic notothenioids revealed extremely high mitochondrial densities [35], [36], [37]. Their liver mitochondria were found highly coupled at low capacities and low levels of proton leakage rates, indicating low costs of mitochondrial maintenance [38], [39]. Recent studies demonstrated some thermal plasticity with elevated capacities of respiratory chain components upon warm acclimation of Antarctic eelpout that indicate different patterns of warm acclimation and the use of metabolic pathways different than those of temperate fish [23].

Less is known, however, about the biochemical mechanisms that shape adaptation of mitochondrial respiratory activity to low and stable temperatures. The effects of temperature on oxygen consumption rates and on the coupling efficiency between electron transport and ATP synthesis in mitochondria of *Trematomus bernacchii* revealed an unusual sensitivity to temperature in line with strong adaptation to cold and high stenothermy at the whole organism level [26]. How this high level of mitochondrial adaptation evolved, has only rarely been studied. In this context, a translocation of the mitochondrial genes encoding NADH dehydrogenase subunit 6 (*ND6*) and the adjacent tRNA^Glu^ has recently been reported [40], [41] for some high-Antarctic notothenioids, with an unclear functional background. *ND6* is seen as an indispensable part of complex I (CI, NADH dehydrogenase; or NADH∶quinone oxidoreductase; EC 1.6.5.3) of the mitochondrial electron transport system (ETS). Complex I is the largest (∼1 MDa) and least understood component of the ETS [42], which provides about 40% of the proton-motive force required to synthesise ATP in vertebrates [43]. Only 7 of the approximately 45 constituent subunits are encoded by mitochondrial genes, namely *ND1*, *ND2*, *ND3*, *ND4*, *ND4L*, *ND5* and *ND6* [44]. Complex I deficiencies and mutations can lead to numerous severe diseases in humans [45], for example, in Parkinson's disease. In zebrafish, the most common gene mutation during onset of Parkinson in humans leads to a reduced function of complex I [46]. Especially *ND4* and *ND6* are essential subunits that ensure correct integration of other subunits into complex I [47], [48], [49]. The translocation of the *ND6* and tRNA^Glu^ genes appears to have occurred only in the more derived five high-Antarctic notothenioid families, among those the Nototheniidae, whereas the basal non-Antarctic families (e.g. Eleginopidae) possess the canonical mitochondrial genome arrangement as commonly found in fish [40], [41]. Along with the translocation of the *ND6* gene, the amino acid sequence of *ND6* has been under evolutionary selection and changed considerably [41]. It has therefore been speculated whether complex I in notothenioid fish was functionally modified [40] or impaired after the translocation of the *ND6* and tRNA^Glu^ genes [41]. Zhuang and Cheng [41] detected signals indicating positive selection within control regions containing the translocated *ND6* gene in the Antarctic notothenioid clade, and selection on several residues within *ND6* genes, suggesting diversifying adaptive change of the protein. They suggested *ND6* modifications may (I) improve protein conformation and therefore complex I subunit interactions at subzero temperatures, and/or (II) a role in modulating mitochondrial complex I redox potential and reactive oxygen species (ROS) production. As ROS production has been attributed to complex I [50], [51], the down regulation of complex I activity under thermal stress could alter reactive oxygen species production. The functional consequences of positioning and the structural change in nototheniid *ND6* have never been analysed, and therefore form the central focus of this study.

We addressed the function and thermal sensitivities of the ETS with particular emphasis on the relative contributions of complex I and complex II (CII, succinate dehydrogenase; EC 1.3.5.1) and the enzymatic capacities of selected proteins in two Antarctic nototheniids. At different temperatures from 0 to 15°C, we measured the oxygen consumption rates and membrane potential in isolated liver mitochondria respiring on several substrates. In addition, we determined enzymatic capacities (NADH dehydrogenase; cytochrome c oxidase, COX) within membrane fraction enriched protein extracts. We further compared the amino acid content of eight notothenioid and non-notothenioid fish species from sub-Antarctic, temperate, tropical and Arctic waters in order to identify possible differences that may provide notothenioid *ND6* with unique biochemical properties and underline their adaptation to the cold.

As behavioural and morphological differences between species can also relate to mitochondrial plasticity and capacities, we compared two endemic and sympatric Antarctic nototheniid species from King George Island (South Shetland Islands), *Notothenia coriiceps* (yellowbelly rockcod) and *Notothenia rossii* (marbled rockcod). Both species have a wide circum-Antarctic distribution, particularly in shelf areas of the Scotia Arc [52], extending to the Antarctic continental shelf in the case of *N. coriiceps*. Functional capacities may differ according to lifestyle requirements [53], which in turn contribute to defining the width of the thermal tolerance window [54]. The two selected species show different adaptations to life in the water column or in benthic habitats, in line with their respective external morphologies: *N. coriiceps* is demersal and sedentary [55], undergoes winter dormancy associated with metabolic suppression [56], and feeds mainly on benthic organisms. *N. rossii* is semipelagic and migratory, and in addition feeds on water column prey during the summer months [57], [58].

## Materials and Methods

### 2.1 Ethics statement

All work on fish was carried out according to the ethics and guidelines of German law. The experiments in this study have been approved according to § 8 Tierschutzgesetz (18.05.2006; 8081. I p. 1207) by the ethics committee of the Senatorin für Arbeit, Frauen, Gesundheit, Jugend und Soziales, Abt. Veterinärwesen, Lebensmittelsicherheit und Pflanzenschutz, Bahnhofsplatz 29, 28195 Bremen, Germany, under the permit number Az.: 522-27-11/02-00 (93) on Jan 15th, 2008 (permit valid until Jan 14th 2012).

### 2.2 Animal capture and handling

Specimens of *Notothenia coriiceps* (Richardson) and *Notothenia rossii* (Richardson) were caught in Potter Cove, Isla 25 de Mayo/King George Island (62°14′S; 058°41′W) by means of baited traps and trammel nets operated from rubber boats in February and March 2009. The traps were 124 cm long, 64 cm wide and 56 cm high, the mesh size was 25 mm, and the opening 240 by 100 mm wide. Trammel nets were 15 m long, the inner mesh was 25 mm. Traps were deployed in depths ranging from 5 to 25 m, water temperature was 1.72±0.13°C and salinity 34.03±0.07 throughout this period. Fish ranged between 30.0 and 36.5 cm standard length (mean: 33.75±2.8 cm, all errors presented as standard deviation of the mean, SD) and 856±251 g weight for *N. coriiceps* and between 30.0–36.5 cm standard length (33.0±2.1 cm) and 601±100 g weight for *N. rossii*. Species were identified morphologically [59].

Fish were kept in several flow-through aquaria systems of about 600 l at Dallmann Laboratory facilities, Jubany Base, King George Island, at 1.0±0.5°C, >90% O_2_ saturation and ambient seawater salinity for at least one week before experimentation.

The animals (n = 10 for each species) were anaesthetised with 0.5 g/l tricaine methane sulphonate (MS 222), blood samples were taken from the caudal vein for further analysis and the liver was excised and stored on ice. The animals were then killed by severing their spinal cord behind the head plates. Blood lactate levels were measured in 20 µl fresh blood with an Accutrend Lactate Analyser (Roche Diagnostics, Germany), haematocrit (Hct) was determined with a Hct microcentrifuge (Compur Microspin M1100, Bayer Diagnostic, Germany).

### 2.3 Mitochondrial isolation

After dissection, the liver was cleaned from blood and total liver weight was taken. A small subsample of 100–200 mg liver tissue was instantaneously frozen in liquid nitrogen for later enzymatic essays, the remaining tissue was weighed and washed in 5 ml/g tissue ice-cold wash buffer (80 mM sucrose, 85 mM KCl, 5 mM EGTA, 5 mM EDTA, 50 mM HEPES, pH 7.1 at 20°C). Subsequently, the tissue was put into 5 ml/g ice-cold isolation buffer (wash buffer+1% w/v fatty acid free BSA, 1 µg/ml aprotinin) and finely minced with scissors. The mixture was then put into a 30 ml Potter-Elvehjem glass homogenizer (VWR International, Germany) and slowly homogenised with three strokes at 80 revolutions/minute. The homogenate was centrifuged (1,300 g, 12 min, 2°C), the supernatant collected and the pellet resuspended in ice-cold isolation buffer and homogenized and centrifuged a second time. Supernatants were then joined and centrifuged (10,500 g, 10 min, 2°C). The supernatant was discarded and the pellet resuspended in ice-cold assay buffer (80 mM sucrose, 85 mM KCl, 5 mM KH_2_PO_4_, 50 mM HEPES, 1% w/v fatty acid free BSA, 1 µg/ml aprotinin, pH 7.1 at 20°C) at a dilution of 1 ml/g initial weight. This stock solution was kept on ice and away from light.

### 2.4 Mitochondrial respiration analysis

A duplicate analysis of each mitochondrial extract was conducted in 2 thermostatted perspex respiration chambers of 3 ml volume (World Precision Instruments, Inc., USA), equipped with an adjustable stopper and ports for triphenylmethylphosphonium (TPMP) and reference electrodes, as well as a titration port for metabolites and inhibitors and one for a TX micro-optode (Presens GmbH, Germany). Micro-optodes were used for fluoroptic measurement of *P*O_2_, membrane potential was measured with a TPMP electrode [60] and a Dri-Ref reference electrode (World Precision Instruments, Inc., USA). The electrodes were connected to a PHM220 voltmeter (Radiometer analytical, France). The voltage output was recorded simultaneously with the oxygen traces by means of a PowerLab recording unit connected to a laptop computer running Chart v5.5.6 software (ADInstruments GmbH, Germany).

Measurements were carried out in assay buffer in a volume of 1.5 ml with mitochondrial concentrations adjusted to about 3 mg mitochondrial protein per ml, at 0, 3, 6, 9, 12 and 15±0.1°C, respectively. Mitochondrial membrane potential was measured as mitochondrial proton-motive force according to Brand [60], with an initial addition of nigericin (80 ng/ml) to clamp *Δ*pH to zero and six subsequent additions of TPMP (1 mM stock) to a final concentration of 6 µM.

Initial respiration and potential were recorded and malate and glutamate added to a final saturating concentration of 1 mM and 1.3 mM, respectively, as substrates providing NADH for complex I. Then ADP (final concentration 0.1 mM) was added and state III (stIII, maximum slope) and state IV (stIV, ADP depleted) respiration and potential recorded. After that, complex I was inhibited with 0.01 mM rotenone (state IV_Rot_) and state II respiration (stII) of complex II activated with FADH_2_ provided by the addition of 2 mM succinate. To record state III and IV again, 0.2 mM ADP were added. State IV^+^ was initiated by 1.3 µg/ml oligomycin. Finally, mitochondria were uncoupled with 0.6 µM carbonyl cyanide p-trifluoromethoxyphenylhydrazone (FCCP).

### 2.5 Enzymatic assays

Functional capacities of the NADH∶cytochrome c oxidoreductase (complex I&III) and cytochrome c oxidase (complex IV, COX) were determined in membrane preparations of liver extracts. Proteins were extracted from liver tissue by homogenisation of frozen tissue in 10 vol. ice-cold extraction buffer (30 mM Tris/HCl, pH 7.5, 250 mM sucrose, 1 mM EDTA, protease inhibitor cocktail for animal tissue (Sigma, Germany)) with a glass homogenizer followed by three treatments with an ultra-turrax (IKA Labortechnik, Germany) for 10 s and intermediate cooling in ice water. Cellular debris was removed by 10 min centrifugation at 1,000 g and 2°C. The supernatant was carefully transferred into a new tube, avoiding co-transferring the upper lipid layer present in the liver preparations. The supernatant was then centrifuged at 218,000 g for 45 minutes. The supernatant was removed and tested for residual activities; the pellet (total membrane fraction) was suspended in 1/5 of starting buffer volume.

All enzyme measurements were conducted in a thermostatted spectrophotometer (Beckman, Fullerton, CA, USA) at 0, 3, 6, 9, 12, 15°C. COX activity was determined according to a protocol modified from Moyes et al. [61] with 2–10 µl of membrane suspension in 1 ml containing 20 mM Tris/HCl, pH 8.0, 0.05% Tween 20 and 0.05 mM reduced cytochrome c. The decrease in absorbance at 550 nm through oxidation of cytochrome c (E_550_ = 19.1 mM^1^ cm^1^) was followed over time. The oxidation of NADH by complex I was followed by monitoring the transfer of electrons to oxidised cytochrome c thus representing the overall capacities of complex I and III, based on a protocol by Möller and Palmer [62] in 25 mM imidazole/HCl, pH 7.4, 125 mM sucrose, 2 mM MgCl_2_, 4 mM sodium azide and 80 µM oxidised cytochrome c. The reaction was started by adding 0.2 mM NADH. The increase in absorbance at 550 nm through reduction of cytochrome c was followed over time.

Protein content was measured in all cellular fractions according to Bradford [63] using a bovine serum albumin (BSA) standard. For mitochondrial protein, the protein content of the assay buffer was considered.

### 2.6 Analysis of amino acid composition

For a comparison of *ND6*, *ND2* and *COI* (cytochrome c oxidase I) structures among eight notothenioid and non-notothenioid fish species from sub-Antarctic, temperate, tropical and Arctic waters, amino acid sequences were retrieved from GenBank (Table S1 for GenBank ID and species names). The amino acid compositions and the instability indexes for each species were computed from the protein sequence using the Expasy ProtParam prediction server (http://us.expasy.org/tools/protparam.html, [64]).

### 2.7 Statistics

Statistical analyses of differences among treatments by ANOVA, ANCOVA, regression analysis and Student's t-tests were carried out using Prism v5.0b, InStat v3.0b (GraphPad Software, Inc.) and Sigma Stat v3.5 (Systat Software, Inc.). The specific test used is given in the respective legend. Differences were considered significant if *p*<0.05. All data are presented as means ± standard error of the mean (SEM), unless stated otherwise.

## Results

### 3.1 Animal parameters

At the time of sampling, fish of both species were of the same size and weight range, but displayed different hepatosomatic indices (liver weight/body weight, HSI) of 2.22±0.33% (*N. coriiceps*) and 1.57±0.06% (*N. rossii*), respectively. Condition factors (weight*100/(standard length)^3^; CF) also differed significantly between the species with 2.18±0.1 for *N. coriiceps* and 1.67±0.04 for *N. rossii*. Haematocrit (Hct) was significantly higher in *N. coriiceps* (30.6±1.1) than in *N. rossii* (27.5±0.8; *p* = 0.034). Blood lactate levels were also higher in *N. coriiceps* (1.7±0.2) than in *N. rossii* (1.0±0.1 mM; *p* = 0.004).

### 3.2 Mitochondrial respiration

**Figure 1:**
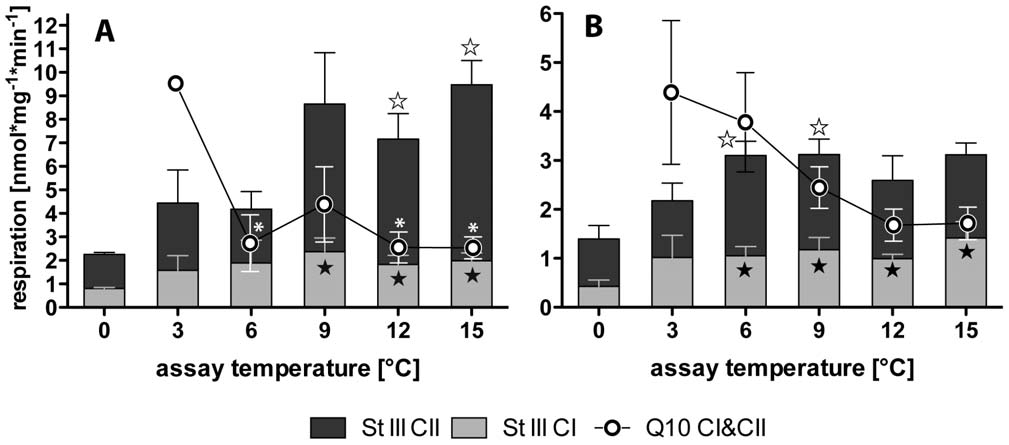
State III respiration and Q_10_ of *N. coriiceps* and *N. rossii* mitochondria. Grey bars represent complex I and black bars complex II respiration for *N. coriiceps* (A) and *N. rossii* (B). Respiration rates significantly different from those at 0°C are indicated by black (complex I) and white (complex II) star symbols. Round symbols indicate Q_10_, calculated between 0°C and the respective temperature. Q_10_ values significantly different from that between 0 and 3°C are indicated by asterisks.

In *N. coriiceps* (fig. 1a) and *N. rossii* (fig. 1b), mitochondrial state III respiration rose with temperature and clearly comprised of contributions by complex I (25–50%) and complex II (50–75%). For *N. coriiceps*, thermal effects on respiration became significant at 9 (CI) and 12°C (CII), respectively, when respiration rates were significantly higher than control respiration at habitat temperature (0°C). In *N. rossii*, mitochondrial respiration reached significantly increased levels already at and above 6°C, which in this species marked the maximum mitochondrial respiratory activity (fig. 1b).

Q_10_ values for total state III respiration were greatest between habitat temperature (0°C) and 3°C (Q_10_ = 9.61±2.39 in *N. coriiceps*; 4.38±1.47 in *N. rossii*), levelling off to values around 3 after 6°C in *N. coriiceps* (significantly lower for 6, 12 & 15°C) and around 2 at 12°C and above in *N. rossii* (fig. 1b). For the whole thermal range of 0–15°C, Q_10_ was 2.60±0.47 for *N. coriiceps* and 1.71±0.33 for *N. rossii*.

**Figure 2:**
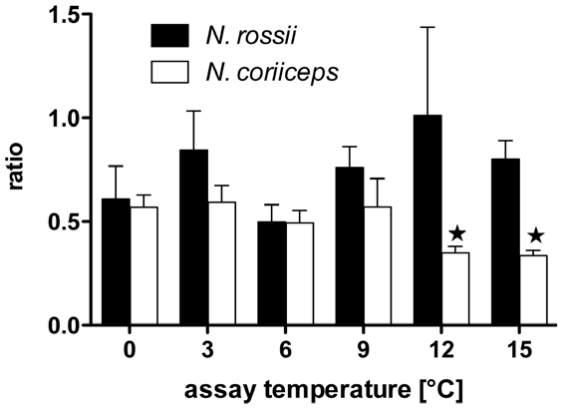
Complex I/complex II ratios of mitochondrial state III respiration for *N. coriiceps* and *N. rossii*. Bars (white for *N. coriiceps*, grey for *N. rossii*) depict means ± SEM, n = 7. Star symbols indicate significant differences from data at 0°C.

Ratios of complex I over complex II contributions to state III respiration are depicted in figure 2. For *N. coriiceps* the ratio was about 0.5 between 0 and 9°C, at temperatures above the ratio decreased significantly, displaying a lower fraction of CI in mitochondrial metabolism at 12 and 15°C. In contrast, *N. rossii* mitochondria showed a slight trend to increasing contributions of CI with rising temperatures.

**Figure 3:**
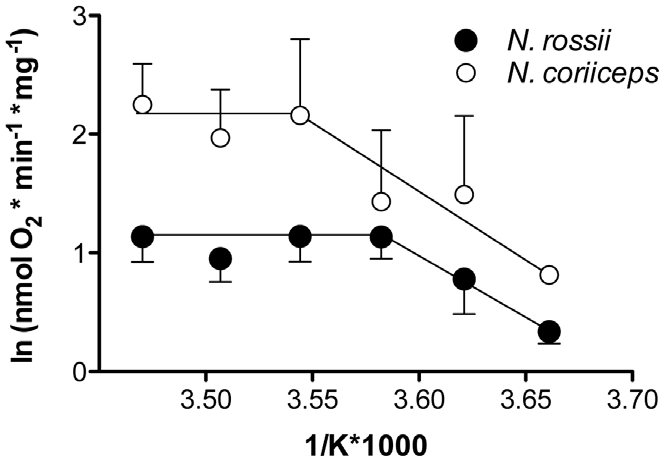
Arrhenius plots of state III respiration of *N. coriiceps* and *N. rossii*. Data are presented as white symbols for *N. coriiceps* and grey symbols for *N. rossii* and represent means ± SEM, n = 7.

Figure 3 illustrates an Arrhenius plot for state III respiration in mitochondria from both *N. coriiceps* and *N. rossii*. In *N.* coriiceps, average activation energy for state III respiration over the whole thermal range was 58 kJ * mol^−1^, starting with 85 kJ*mol^−1^ between 0 and 9°C and then levelling off to about 10 kJ * mol^−1^ between 9 and 15°C in *N. coriiceps*. *N. rossii* showed an overall activation energy of 59 kJ * mol^−1^ (0–9°C), starting with 84 kJ * mol^−1^ between 0 and 6°C and levelling off to about 10 kJ * mol^−1^ between 9 and 15°C.

Respiratory Control Ratios (RCR (stIII/IV)and RCR^+^ (stIII/IV^+^ and stIII/IV_Rot_, respectively)) were stable over the experimental thermal range. Mean RCR^+^ were 5.57±0.33 (CI) and 5.49±1.2 (CII) for *N. coriiceps*; and 4.77±0.59 (CI) and 4.88±0.46 (CII) for *N. rossii* (further RCR data presented in Table S4). Thus, maximum proton leak rates (involving CI and CII) accounted for about 18% of the physiological oxidative capacities in *N. coriiceps*, and for about 21% in *N. rossii*.

Although Acceptor Control Ratios (ACR, the dependence of the O_2_ consumption rate on the P_i_ acceptor ADP, i.e. state III/II) appeared to decrease above 3°C, there are no significant differences in ACR between assay temperatures. Mean ACR for *N. coriiceps* were 2.29 (CI, malate), 2.17 (CI, malate+glutamate) and 2.74 (CII, succinate+rotenone), for *N. rossii* ratios of 1.60 (CI, malate), 1.56 (CI, malate+glutamate) and 1.96 (CII, succinate+rotenone) were estimated.

ADP/O ratios were stable over the thermal range tested (and somewhat higher for complex I; c.f. Table S4), they did not differ significantly between complex I and II, but between species: in *N. coriiceps*, mean ADP/O ratios were 2.44±0.11, in *N. rossii* 1.97±0.15.

### 3.4 Enzymatic capacities

**Figure 4:**
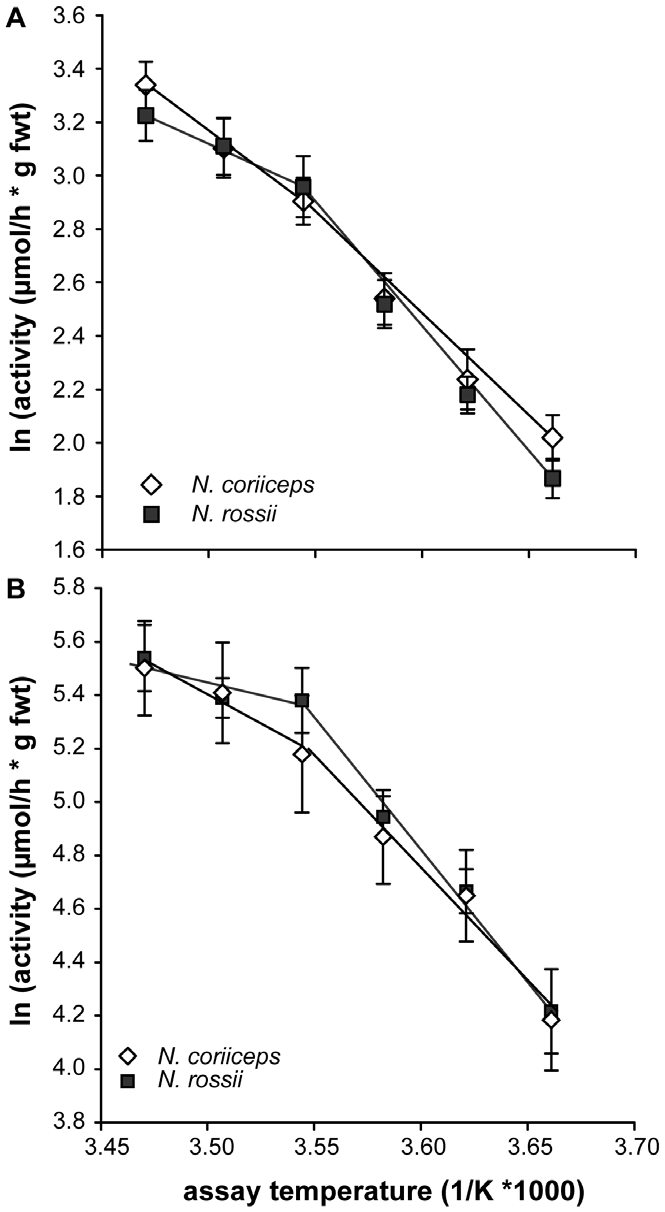
Arrhenius plots for NADH∶cytochrome c oxidoreductase and cytochrome c oxidase of *N. coriiceps* and *N. rossii*. Panel A presents data for NADH∶cytochrome c oxidoreductase, panel B for cytochrome c oxidase (white symbols: *N. coriiceps*, grey symbols: *N. rossii*). Arrhenius break temperatures are located around 9°C (3.54 K*1000^−1^) for both enzymes and species. Data are presented as means ± SEM, n = 10.

**Figure 5:**
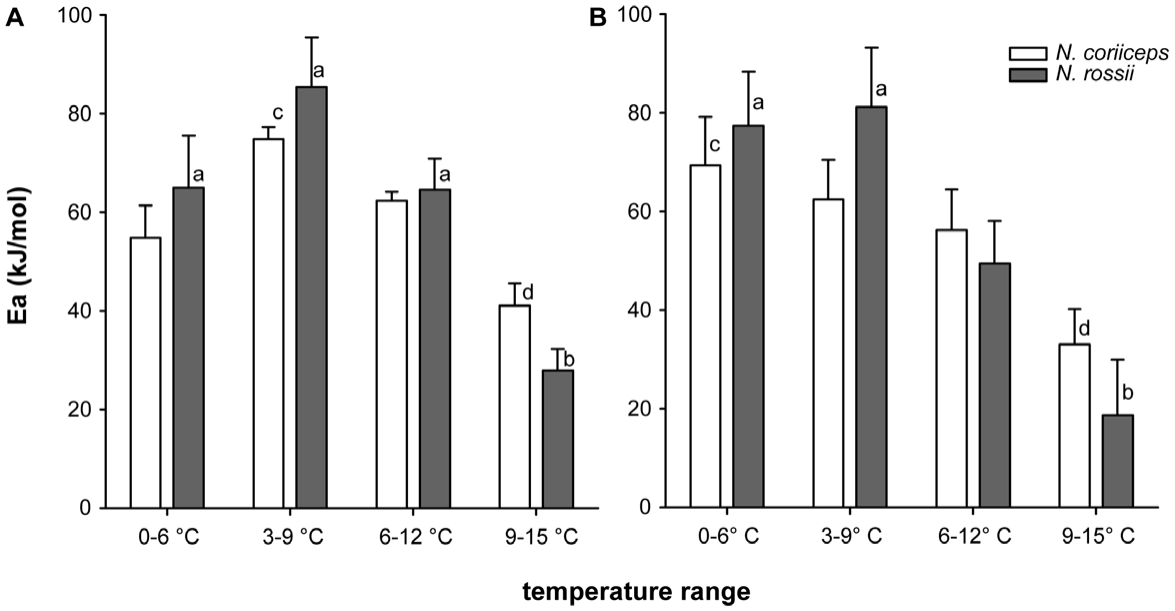
Activation energies for NADH∶cytochrome c oxidoreductase and cytochrome c oxidase of *N. coriiceps* and *N. rossii*. Panel A presents data for NADH∶cytochrome c oxidoreductase, panel B for cytochrome c oxidase (white bars: *N. coriiceps*, grey bars: *N. rossii*). Differences in E_a_ over the thermal range were tested using RM-ANOVA and are indicated by different letters (a/b; c/d). Data are presented as means ± SEM, n = 10.

NADH/cytochrome c oxidoreductase activity showed a strong temperature dependency in both species (Table 1; fig. 4a) ranging from 7.9±0.6 to 29.2±2.5 µmol*h g fwt^−1^ between 0° and 15°C in *N. coriiceps* and 6.7±0.5 to 26.3±2.6 µmol*h g fwt^−1^ between 0° and 15°C in *N. rossii*, respectively. Similarly, cytochrome c oxidase (complex IV) activity rose from 77.4±14.4 and 76.0±11.9 to 280.6±44.6 and 273.0±33 µmol*h g fwt^−1^ between 0° and 15°C in *N. coriiceps* and *N. rossii*, respectively (fig. 4b). No significant differences were found between the species at any assay temperature. The Arrhenius plots revealed discontinuous slopes for both enzymes and species (fig. 4), which became visible in the respective activation energies when compared in steps of 6°C over the thermal range investigated (fig. 5).

**Table 1:** Functional capacities of NADH/cytochrome c oxidoreductase and cytochrome c oxidase.

Species	Temp.	NADH/Cytc ox/red	Cytochrome c oxidase	Ratio
*N. coriiceps*	0	7.9±0.6	77.4±14.4	9.8
	3	9.9±1.0	118.6±18.8	12.0
	6	13.2±1.3	148.7±24.0	11.2
	9	18.9±1.7	214.4±38.8	11.3
	12	23.5±2.5	259.1±43.6	11.0
	15	29.2±2.5	280.6±44.6	9.6
*N. rossii*	0	6.7±0.5	76.0±11.9	11.3
	3	9.1±0.6	109.5±9.1	12.2
	6	12.9±1.3	144.5±11.6	11.2
	9	20.5±2.6	232.6±29.6	11.3
	12	23.6±2.4	225.2±16.6	9.5
	15	26.3±2.6	273.0±33.0	10.4

Activities of both enzyme complexes are given in µmol cytochrome c * h g fwt^−1^. Ratios between NADH/Cyt c ox/red and COX are given in the last column.

In all cases, activation energies were highest between 0 and 9°C and significantly reduced in the range of 9 to 15°C. This drop in thermal sensitivity was more pronounced in *N. rossii* with an about 2 to 3-fold reduction for NADH/cytochrome c oxidoreductase (74.8±8.4 kJ*mol^−1^ to 27.9±4.3 kJ*mol^−1^; fig. 5a) and about 4-fold for cytochrome c oxidase (79.3±9.5 kJ*mol^−1^ to 18.6±11.3 kJ*mol^−1^; fig. 5b; *N. coriiceps*: NADH/cyt c oxred: 74.8±6.2 to 41.0±4.5 kJ*mol^−1^; COX: 65.9±6.2 to 33.0±7.2 kJ*mol^−1^). Due to the higher variability in the measurements at higher temperature, no significant difference between the species could be demonstrated in that range (NADH/Cyt c oxred: *p* = 0.065).

When expressed per µmol cytochrome c, cytochrome c oxidase capacities were found to be about 10- to 12-fold in excess compared to NADH/cytochrome c oxidoreductase in both species at all assay temperatures.

### 3.5 Mitochondrial membrane potential

**Figure 6:**
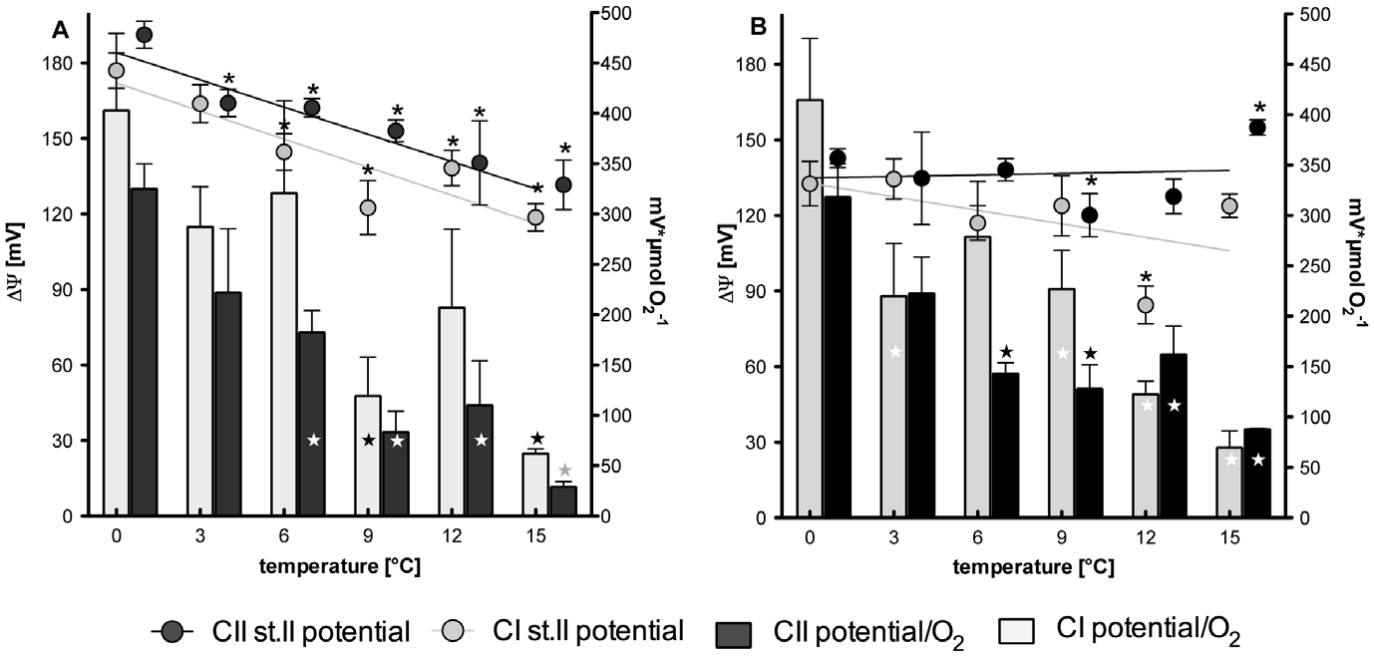
State II membrane potential and potential realised per mol O_2_ consumed for *N. coriiceps* and *N. rossii*. Panel A presents data for *N. coriiceps* and panel B for *N. rossii*. Mitochondrial membrane potential (symbols) is given as *Δ*Ψ (left ordinate) for respiration involving complex I (grey symbols) and respiration involving complex II (black symbols). Bars represent the potential realised per µmol O_2_ consumed (right ordinate), grey bars depict values for complex I, black bars for complex II. Data are presented as means ± SEM, n = 7.

The two panels in figure 6 display both the mitochondrial membrane potentials realised during state II respiration (circles) and the ratio of state II membrane potential over the respective oxygen consumed (bars); CI and CII were analysed separately.

Membrane potentials decreased significantly with temperature in *N. coriiceps* (CI *p* = 0.011, slope: −3.7±0.8; CII *p* = 0.001, slope: −3.6±0.5) but not so in *N. rossii* (CI *p* = 0.26; CII *p* = 0.86; fig. 6, round symbols). In *N. coriiceps* potentials were significantly reduced beyond 3 (CII) and 6°C (CI), respectively (fig. 6a). In *N. rossii*, the membrane potential remained rather stable, it was significantly reduced only at 9°C in CII and at 12°C in CI, and significantly increased at 15°C in CII (fig. 6b).

The membrane potential generated per mole of oxygen consumed in state II (solid bars in fig. 6 A, B) decreased with temperature to a similar extent in both species and for both complexes (*N. coriiceps*: CI *p* = 0.016, slope: −20.4±5.1; CII *p* = 0.002, slope: −18.2±2.7 and *N. rossii*: CI *p* = 0.011, slope: −19.7±4.4; CII *p* = 0.022, slope: −12.9±3.5). In *N. coriiceps* (fig. 6a), the ratios of potential/O_2_ were significantly reduced at 9 and 15°C in CI and from 6 to 15°C for CII. *N. rossii* showed significant reductions in potential/O_2_ at 3°C and between 9 and 15°C for CI and from 6 to 15°C for CII (fig. 6b). As decreasing potential/O_2_ ratios can mainly be attributed to increasing electron flux with temperature, Q_10_ values for membrane potential/O_2, stII_ and 1/O_2, stII_ were compared by t-test for both complexes and species (0–15°C) to identify whether decreased efficiencies to generate high membrane potentials during warming further reduced the ratios of potential/O_2_. In *N. coriiceps*, decreasing membrane potentials significantly contributed to a further reduction in potential realised per mol O_2_ consumed in CII (individual Q_10_ data presented in Table S4). This implies that mitochondrial metabolism could not be increased with rising temperature to an extent to keep state II membrane potential constant.

### 3.6 Analysis of amino acid composition

The instability indices presented in table 2 provide protein instability estimates based on a statistical analysis of amino acid composition [65]. A protein with an instability index less than 40 is considered stable, whereas values above 40 predict instability. For *ND6* (Table 2, first column), it is apparent that the three Antarctic species and the sub-Antarctic notothenioid have indices close to or above 40, while the Arctic, temperate and tropical species are significantly below this critical threshold (t-test, p = 0.002). *ND2* displays a high instability in all the species analysed (Table 2, second column), owing to a much more similar amino acid composition (data not shown, for further information refer to Table S2). *COI* appears to be much more stable, with lower instability indices throughout (Table 2, third column, and Table S3).

**Table 2:** The table reports for each species the instability indices of the *ND6*, *ND2* and *COI* proteins computed using Expasy's ProtParam (http://us.expasy.org/tools/protparam.html) prediction server [64].

*Species*	Instability index *ND6*	Instability index *ND2*	Instability index *COI*
***Notothenia coriiceps***	39.19	38.48	28.23
***Notothenia rossii***	38.92	38.17	22.64
***Pachycara brachycephalum***	38.29	37.29	24.75
***Eleginops maclovinus***	40.92*	42.38*	24.87
***Boreogadus saida***	21.07	39.55	24.61
***Arctogadus glacialis***	20.58	38.95	24.61
***Gadus morhua***	20.58	39.30	24.61
***Chlorurus sordidus***	32.83	35.51	25.60

* protein classified as unstable (threshold: 40).

## Discussion

### 4.1 Animal parameters

Animal data indicate that stress levels were generally low and the experimental animals in good condition, comparable to specimens caught regularly in Potter Cove and its direct vicinity [57], [66]. In *N. coriiceps* of the same size class and area, Kamler and colleagues [67] found similar HSI of 2.6±1.0 and condition factors of 2.4±0.3.

In *N. coriiceps* that had been transported to and kept in the UK, Egginton [68] found somewhat lower Hct (16–19%) and lactate levels (0.05–0.4 mM) than presented here. Heise and Abele [69] reported similar Hct (24%), but much higher lactate levels (around 7.5 mM) for *N. coriiceps* from the same site, while Beers and Sidell [70] even found a Hct of 35% and lactate levels around 1 mM around the Western Peninsula. At Signy Island, *N. coriiceps* and *N. rossii* display haematocrits of 24–25% [71]. This variability, especially in lactate concentrations, may be due to (long-distance) transportation and handling stress, yet it generally shows that the genus Notothenia does produce lactate, despite the reduced glycolytic capacities found by Dunn & Johnston [72].

The differences in hepatosomatic indices and condition factors, both of which were higher in *N. coriiceps* (cf. 3.1), relate to the morphological and physiological differences consistent with the differential adaptations to inhabit the water column of the benthic *N. coriiceps* and the bentho-pelagic *N. rossii*. This becomes evident by a greater density, expressed as mean percentage buoyancy of *N. coriiceps* (4.34%) over *N. rossii* (3.82%) [73]. *N. coriiceps* is a heavy rugged fish, and field observations with underwater cameras showed that it is a rather inactive sit-and-wait predator [55]. In comparison to *N. rossii*, *N. coriiceps* has a greater weight per unit length that is associated with a thicker body; skeletal weight as a percentage of body weight was also significantly greater in *N. coriiceps* (2.46%) than *N. rossii* (1.65%; *t* = 5.611, *P*<0.03) [73]. The more active *N. rossii* is more gracile with a laterally compressed and streamlined body. Its vertebrae morphology consists of bone that is more spongy or porous and of neural arches thinner than those of *N. coriiceps* [73]. *N. rossii* occurs in Potter Cove from 0 to 6–7 years exclusively in the juvenile stage [74], [75], after which they migrate offshore to join and spawn with the adult population [76]. Data on age, gonadal stages and depth distribution of *N. coriiceps* at Potter Cove suggest that sexual maturity is first reached at about 6 years and that the species may remain near shore during its whole life cycle [57]. With regard to whole animal performance, it is therefore not surprising that Egginton [68] found a more pronounced drop in arterial *P*O_2_ and higher lactate levels during and after exhaustive exercise in *N. coriiceps* compared to *N. rossii*.

### 4.2 Mitochondrial function

This study provides evidence for the functional integrity of complex I in the Antarctic nototheniids *N. coriiceps* and *N. rossii*, despite the translocation of *ND6*. To our knowledge, only a few studies presently exist that investigate complex I function individually in fish (let alone the consequences of genetic defects), yet it has been reported by Hilton and colleagues that in triplefin fishes CI contribution decreases with temperature from about 50 to 30% [77]. In sea bass (*Dicentrarchus labrax*), complex I contribution is around 30–40% (Mark et al., in prep). In this light, complex I/II ratios of 0.3 to 1.0 point at a regular contribution of complex I to total mitochondrial energy metabolism.

Mitochondrial respiration in state III (in figs. 1 expressed as the sum of the maximal capacities of complex I and II) at 0°C were around 2.2±0.1 and 1.4±0.4 nmol O_2_*min mg protein^−1^ for *N. coriiceps* and *N. rossii*, respectively. Hardewig and colleagues [27] found 3.7±1.2 nmol O_2_*min mg protein^−1^ for *L. nudifrons* liver mitochondria, complex I inhibition with rotenone (8 µM) resulted in 50% respiration reduction, even during respiration on succinate (3.3 mM) alone. Johnston and colleagues reported higher values for red muscle mitochondria at 0°C, in *N. coriiceps* they found 7.7 [78], in *L. nudifrons* 11.8±6 nmol O_2_*min mg protein^−1^ (−1°C, [36]) and in the sub-Antarctic *E. maclovinus* (4°C) 24.2±3.5 nmol O_2_*min mg protein^−1^. All these data fall in the same range when taking into account that there are capacity differences between liver and red muscle mitochondria [79], which in goldfish results in an about two-fold discrepancy in favour of muscle tissue [80].

At 0°C, mitochondrial respiration of *N. rossii* was up to 30% lower than in *N. coriiceps*, this may relate to the generally higher respiration rates in *N. coriiceps*: Ralph & Everson [81] estimated whole animal metabolic rates to be 1.75 mmol*kg h^−1^ in *N. coriiceps* and 1.19 mmol*kg h^−1^ in *N. rossii*, reflecting the ratio of mitochondrial capacities.

However, full state III capacity under CI and CII substrates and saturating ADP (denoted OXPHOS) is non additive. CI and CII analysed individually (CI: NADH related substrates; CII: succinate & rotenone) only reach up to about 70 and 80% of total OXPHOS, respectively. This is a frequent finding (in human muscle fibres, there is an approx. 35% discrepancy between OXPHOS capacities and the sum of CI & CII capacities) and may be caused by a downstream limitation in mitochondrial complex III (Cytochrome c Reductase) and/or complex IV (Cytochrome c Oxidase), after the Q cycle where the branches of the ETS converge. Further substrate oxidation in the Krebs cycle (after succinate dehydrogenase) that yields more NADH is also unlikely due to diffusive loss of Krebs cycle products out of the mitochondria [82]. Following analysis of complex I and II capacities individually, simple addition of individual fluxes will overestimate maximal OXPHOS capacities.

### 4.3 Mitochondrial metabolism

In both species, the decrease of state III Q_10_ values indicates decreased mitochondrial scope beyond 6 (*N. coriiceps*) and 9°C (*N. rossii*), respectively. Similar values reported for *L. nudifrons* [27] and *N. coriiceps* red muscle mitochondria [78] at elevated temperatures corroborate these findings. High Q_10_ values between 0 and 9°C are mirrored by high Arrhenius Activation Energies (E_a_) for mitochondrial state III respiration, which fall to much lower E_a_ values beyond 9°C. This is typical for Antarctic animals, values of around 60 kJ*mol^−1^ have been found in gill mitochondria of the Antarctic bivalve *Laternula elliptica* [39] between 0 and 3°C, which are similar to the values found in liver mitochondria from the notothenioid *L. nudifrons* (47.5 kJ*mol−1, [27]) or red muscle mitochondria from *N. coriiceps* (73 kJ*mol−1, calculated from data presented in [83], between −1.5 and −2.5°C). At temperatures above 5°C, Weinstein & Somero [26] already observed a decrease of E_a_ in the notothenioid *Trematomus bernacchii* (38.3 kJ*mol^−1^).

The transition from high to low E_a_ is sharp in *N. rossii* and *N. coriiceps* and is characterised by an Arrhenius Break Temperature (ABT, fig. 3) at 6 and 9°C, respectively. These are similar to *L. elliptica* [39]. While the E_a_ differed between *N. rossii* and *N. coriiceps*, the activation energies before and after the ABT similar for the two fish species (cf. 3.2, fig. 3). This cannot be quantified more precisely here, as at present there are no data available for temperatures between 6 and 9°C. ABTs can be assumed to be closer to 9 than to 6°C. In some contrast to the findings presented here, mitochondrial state III ABTs occurred above 15°C [27] and beyond 20°C [26] in the Antarctic nototheniids *L. nudifrons* and *T. bernacchii*, respectively.

Mitochondrial capacities showed a clear thermal limitation, in that increased temperatures led to decreased membrane potentials and no further increments in respiration rate beyond a certain thermal threshold characterised by the ABT. At first sight, this limitation might contribute to setting the whole organism *pejus* temperature, where capacity limitations set in [84]. Yet, due to their lower level of organisational complexity, thermal tolerance windows of organelles generally span a wider temperature range than those of the whole organism [25]. Notably however, Bilyk & De Vries [18] and Beers & Sidell [70] found acute critical thermal maxima (CT_max_) for *N. coriiceps* and *N. rossii* around 16–17°C (when acutely warmed from −1°C by 0.3°C*min^−1^ and 3.6°C*hour^−1^, respectively). Chronic heat tolerance limits of Antarctic fish are found at much lower temperatures: the Antarctic nototheniid *Pagothenia borchgrevinki* displays first cardiac limitations when acutely warmed to 6°C [85] and has been shown to be able to adapt to a chronic exposure of 4°C [17], [20]. *N. rossii* can be acclimated to up to 7°C for several weeks (authors' personal observations), thus the *pejus* range for Antarctic nototheniids can be assumed to be generally located between 4 and 9°C, with critical temperatures located beyond 7–8°C for *N. coriiceps* and *N. rossii*.

Nonetheless, mitochondrial efficiency appears to be safeguarded in the two nototheniids: RCRs were stable over the experimental temperature range, indicating rather static mitochondrial leak rates independent of temperature. Hardewig and colleagues [27] as well as Johnston and colleagues [78] report RCR^+^ values between 7 and 10 for Antarctic notothenioids, which correspond to apparent proton leak rates of 10–15%. The mean leak rates observed for liver mitochondria of the two nototheniids in this study were only slightly higher than these values (18–21%).

As a possible consequence of stable RCR^+^, ADP/O ratios also remained unchanged over the thermal range in this study in both nototheniids. They were higher than the values reported by Hardewig and colleagues [27] for *L. nudifrons* (around 1.5), but similar to those observed in short-horn sculpin *M. scorpio* [86] and rainbow trout [87]. In the range of 0–15°C, both complexes display ADP/O ratios similar to or even higher than active temperate fish species, especially so in *N. coriiceps*. In terms of ADP generation, complex I can be thus assumed to be as efficient and thermally stable as complex II in the two nototheniids.

### 4.4 Enzymatic function

Function of the mitochondrial complexes, as evidenced by the enzymatic assays of complex I/III and complex IV, mirrored (and thus corroborated) the results of the respiratory studies in isolated mitochondria. As observed for mitochondrial state III respiration (cf. 3.2), ABTs were located around 9°C (fig. 4) and activation energies were highest in the range from 0–9°C in both enzymatic complexes and species (fig. 5). E_a_ of the two enzyme complexes (66–80 kJ*mol^−1^) were very close to the activation energies observed in mitochondrial state III respiration (84 kJ*mol^−1^, cf. 4.3), which can be taken as a sign of good mitochondrial coupling. Activation energies for COX were also higher than in temperate fish: in Mediterranean sea bass (*D. labrax*), E_a_ for COX in the range of 3–20°C is between 10–13 kJ*mol^−1^ (depending on acclimation, [88]), whereas in cold acclimated temperate eelpout *Z. viviparus* [89] values were around 35 kJ*mol^−1^ (similar to those found in *Notothenia sp.* between 9–15°C).

Higher values (84 kJ*mol^−1^) thus appear typical for Antarctic fish and corroborate the general concept of higher activation energies of mitochondrial enzymes in cold-adapted ectoterms [27], [39], [90]: mitochondrial densities are increased in the cold putatively to shorten diffusion distances [91], as a consequence, the activities of the resulting high number of mitochondrial enzymes have to be kept at a physiological level by increasing activation energies accordingly, especially in stenotherms. This becomes evident by the total activities for COX: with values of around 80 µmol * h g fwt^−1^, they are in the same range or even lower than those in the Antarctic eelpout *P. brachycephalum* (around 210 µmol*h g fwt−1, [89]), in liver of temperate cod *G. morhua* (8°C) (90–200 µmol*h g fwt−1, [92]), or in temperate and Arctic cottids and zoarcids (1°C) (120–180 µmol*h g fwt−1, [93]).

### 4.5 Energetic coupling of complex I

When compared on the basis of cytochrome c turnover, complex I/III (NADH/cyt c ox/red) displayed a 10-fold lower activity than complex IV (Cyt c oxidase). Under *in vivo* conditions, complex II, flavines and glycerophosphate dehydrogenase (GpDH) also contribute electrons via the ubiquinone pool to complex III. Complex III activity was hence limited to maximal complex I activity in the protocol used here and presumably would have shown higher capacities under further electron contributions from complex II (and perhaps GpDH). Secondly, excess capacities are frequently found downstream in the ETS, which for complex IV can be quite dramatic in invertebrates [94], and have been found to be at least 2.5–3 fold with respect to OXPHOS capacities in triplefin blennies [77]. Complex IV is a potential rate-limiting enzyme in the ETS [95] and acts as an electron sink. High activities of CIV can effectively elevate the mitochondrion's affinity for O_2_ [96].

Finally, the different activities may also reflect a decreased CI capacity: expressed as protons pumped into the intermembrane space per pair of electrons translocated (or per mol O consumed), the theoretical stoichiometry between complex I and II is 10∶6 (complex I and III pump 4 protons each, complex IV pumps 2; c.f. 43). Therefore, at 4 protons per ATP (3 for synthesis, 1 for translocation by the ANT), this translates into 2.5 vs 1.5 ATP per pair of electrons, or an ADP/O ratio of 2.5 for complex I and 1.5 of complex II. The ADP/O ratios found in this study are not fully concordant with these theoretical values. In both species, ADP/O were only slightly higher for complex I than for complex II and under OXPHOS respiration in state III, complex I respiration only equals, or is even smaller than that of complex II, which comprises 50–75% of mitochondrial respiration (fig. 2). Although one may interpret this as defects in complex I, mean ADP/O ratios approaching 2.5 (*N. coriiceps*) and 2.0 (*N. rossii*) may not indicate ineffective phosphorylation rates but differences in electron flow from complexes I and II and convergence on complex III (cf. 4.2).

The efficiencies of complex I and II can be compared on basis of their ability to generate membrane potential: For every pyruvate that enters the Krebs cycle, 4 NADH are oxidised by complex I and 1 succinate is oxidised by complex II, resulting in 40 protons (10 ATP) and 6 protons being pumped (1.5 ATP) by the respective complexes. In terms of ATP production, complex I is 6 times more efficient than complex II (10 vs. 1.5 ATP) and should contribute 4 times more to the overall mitochondrial phosphorylation capacity than complex II (4 vs. 1 pair of electrons). As these contributions cannot be differentiated by respiration analyses alone, measurement of membrane potential in leak respiration states (i.e. in state II) theoretically should reveal that complex I was 1.66 times more effective at generating membrane potential than complex II (proton stoichiometry of 10∶6).

Figure 6 depicts these ratios of membrane potential per state II oxygen consumption (bars) and generally, complex I shows the expected pattern with mean ratios of complex I *vs.* II of 1.62±0.14 (*N. coriiceps*), and 1.26±0.21 (*N. rossii*). Again, *N. coriiceps* presents a fully functional complex I, operating close to the theoretical optimum. However, these ratios are lower and decrease further with rising temperature for *N. rossii*.

A decrease of complex I function with rising temperature has been reported for triplefin blennies [77] and supports the concept of a thermally sensitive, but otherwise properly working complex I. There are few studies comparing complexes I and II in ectotherms at different temperatures and it is therefore not possible to compare our data to further fish species with the typical canonical gene order for *ND6*. It is clear that complex I has a relatively high thermal sensitivity, especially so in *N. rossii*, which may result from structural peculiarities of notothenioid *ND6*.

### 4.6 *ND6* structure

The protein instability indices (Table 2) underline the general notion that decreased thermal stabilities of cold-adapted enzymes are the side effects of an increased flexibility, which is considered a precondition for proper function at low temperatures [97] and may also have been a pre-adaptation for the Antarctic notothenioid lineages to radiate into the Southern Ocean (even before *ND6* translocation). Modifications to increase flexibility may include a decrease in weak interactions and hydrophobicity, as well as substitution and deletion of specific amino acids [98]. In this respect, nototheniid *ND6* may not only have undergone a translocation, but also some changes in composition. Table 3 lists the percentages of the individual amino acids in *ND6* (for *ND2* and *COI* amino acid composition, refer to Tables S2 and S3, respectively). In fact, there are not only composition differences between the Notothenioid/eelpout and temperate/Arctic/tropical (i.e. non-Antarctic) group but also between the two nototheniid species (*N. coriiceps*, *N. rossii*) and the related sub-Antarctic notothenioid *E. maclovinus* (Eleginopidae). *E. maclovinus* has been described as ‘notably divergent from the rest of the notothenioids’ in terms of protein composition [99], which appears to carry characteristics of both groups in its *ND6* composition: the 3 Antarctic species and the sub-Antarctic notothenioid bear lower leucine contents than the non-Antarctic groups, but higher percentages of cysteine, which is even more prominent in the nototheniids (*ND6* translocated). All notothenioids lack histidine in their *ND6* structure, the Antarctic nototheniids possess lysine, which is not found in any of the species with canonical gene order. Glutamine also is only present in the Antarctic group (including the zoarcid), with a fourfold difference between nototheniids and the species with canonical gene order. These changes in amino acid composition may be indicative of cold adaptation in the notothenioids and the Antarctic eelpout, rendering *ND6* more flexible at cold temperatures but in turn also bring about a higher thermal sensitivity of the protein. This flexibility becomes evident in the instability indices, also reported in Table 2 for *ND2* and cytochrome c oxidase I (*COI*). *ND2* displays similarly high instability indices as *ND1*, thereby corroborating the commonly observed high thermal sensitivities of complex I [77]. Complex IV (*COI*) on the other hand, is thermally very stable, has high Q_10_, low instability indices (cf. Table 2) and is generally found to be very much conserved throughout the animal kingdom (and is therefore often used for phylogenetic analyses). Of the three proteins analysed, only *ND6* showed significant differences in instability indices between the nototheniids and the other species, which may indicate a further cold-adaptation of this protein. It remains unclear, however, whether cold-adaptation only became possible with the translocation of the *ND6* gene.

**Table 3:** Amino acid composition of *ND6*.

	Ala	Arg	Asn	Asp	Cys	Gln	Glu	Gly	His	Ile	Leu	Lys	Met	Phe	Pro	Ser	Thr	Trp	Tyr	Val
***N. coriiceps***	10.3	1.7	0.6	0.6	**4.0**	**2.3**	4.6	13.8	*0.0*	2.3	**14.9**	**1.1**	5.7	5.2	3.4	8.0	2.9	1.7	4.6	12.1
***N. rossii***	10.9	1.7	0.6	1.1	**4.0**	**2.3**	4.0	13.8	*0.0*	2.3	**14.4**	**1.1**	6.3	4.6	3.4	8.0	2.9	1.7	4.6	12.1
***P. brachycephalum***	11.1	3.5	0.6	1.8	2.3	0.6	4.7	14.0	**0.6**	4.7	**14.6**	*0.0*	1.2	5.8	4.1	7.6	2.3	2.9	4.7	12.9
***E. maclovinus***	7.5	1.7	0.6	1.2	2.3	0.6	4.0	15.0	*0.0*	2.9	**14.5**	*0.0*	6.4	8.1	2.9	8.7	2.9	0.6	5.2	15.0
***B. saida***	11.0	1.7	0.6	1.2	*1.2*	*0.0*	4.0	14.5	**0.6**	2.9	*20.2*	*0.0*	4.0	3.5	2.9	6.9	1.7	4.0	4.6	14.5
***A. glacialis***	11.6	1.7	0.6	1.2	*1.2*	*0.0*	4.0	14.5	**0.6**	2.9	*19.7*	*0.0*	4.0	3.5	2.9	6.9	1.7	4.0	4.6	14.5
***G. morhua***	11.0	1.7	0.6	1.2	*1.2*	*0.0*	4.0	14.5	**0.6**	2.9	*19.7*	*0.0*	4.0	3.5	2.9	6.9	1.7	4.0	4.6	15.0
***C. sordidus***	13.3	2.3	0.6	2.3	*0.6*	*0.0*	3.5	12.7	**0.6**	3.5	*18.5*	*0.0*	2.9	5.8	2.9	5.8	2.9	2.9	5.8	13.3

The table reports for each species the amino acid composition of the *ND6* protein computed using Expasy's ProtParam (http://us.expasy.org/tools/protparam.html) prediction server [64].

### 4.7 Conclusions

In the light of the translocation of the *ND6* gene, we focused our interest on the performance and capacity of the individual mitochondrial complexes I and II. We measured mitochondrial performance during warming, in two stenothermal nototheniid fish species that show slightly different distribution, ecology and life histories.

By specific analysis of CI efficiency, here we demonstrate that despite *ND6* translocation CI remains functional and well coupled. For *N. coriiceps* CI coupling appears to be greater for *N. rossii*, and this is most apparent in complex I ADP/O ratios. These indicate a generally better mitochondrial performance of *N. coriiceps*, which was observed in most parameters that were investigated in this study. Only state II membrane potential (fig. 6) was found to be more stable over the thermal range in *N. rossii*, pointing at a somewhat tighter inner mitochondrial membrane or better coordination and thermal stability of the enzymes involved into the generation of membrane potential.

Overall, mitochondrial thermal responses were similar in both nototheniids: in mitochondrial respiration (and all resulting ratios, figs. 1 & 3) and enzymatic function (figs. 4 & 5), capacities increased until close to 9°C, above which respiration and enzymatic activity levelled off. This break, as characterised by the ABT, was more pronounced on the mitochondrial level (fig. 3) than in the enzymatic complexes analysed individually. This is in line with the general notion that higher degrees of functional integration bring about higher thermal sensitivity [30]. *Notothenia coriiceps* generally displayed a higher amplitude in thermal response (figs. 1 & 3), while *N. rossii* showed higher enzymatic E_a_ (fig. 5) and in part also higher enzymatic activities (fig. 4, COX). ABTs may be found at slightly lower temperature in *N. rossii* (cf. fig. 3), however greater resolution between 6 and 9°C is required. These differences in mitochondrial metabolism between the two species could also be regarded as a trade-off for different liver sizes (HSI) and compositions (elevated fats) and ontogeny rather than as a sign of differential thermal adaptation between the two species (cf. 4.1). The low thermal tolerance thresholds in terms of ABT values presented here for nototheniid mitochondria most likely reflect the trade-offs in cold adaptation of mitochondrial proteins, in that increased flexibility at very low temperatures go hand in hand with reduced thermal stability, as demonstrated above for *ND6*.

In the light of the present study, one has to ask for the long-term perspective for the two species under the current scenarios of global warming (and ocean acidification), which are particularly dramatic along the Antarctic Peninsula [100], [101], [102], [103]. Some physiological adjustments after warm acclimation to 4°C have been found in the cardiovascular system [16], [19], [20], [104] and the metabolic rate of the cryo-pelagic Antarctic nototheniid *Pagothenia borchgrevinki* [17]. At the mitochondrial level, there is further evidence for limited acclimation capacities in the Antarctic eelpout *P. brachycephalum* [21], [23], [105]. Whether mitochondrial metabolism in *N. rossii* and *N. coriiceps* will be able to similarly adapt and how molecules, organelles and cells in general will respond to long-term environmental changes remain important and stimulating topics for future studies.

## Supporting Information

Table S1 **ND6, ND2 and COI amino acid sequences compared in this study.** The table reports: species names, general description of habitat, protein (aa) sequence GenBank accession number for each protein and reference.
(DOC)

Table S2 **Amino acid composition and instability index of the ND2 protein.** The table reports for each species the amino acid composition of ND2 protein and the instability index computed using the Expasy's ProtParam (http://us.expasy.org/tools/protparam.html) prediction server [64].
(DOC)

Table S3 **Amino acid composition and instability index of the COI protein.** The table reports for each species the amino acid composition of COI protein and the instability index computed using the Expasy's ProtParam (http://us.expasy.org/tools/protparam.html) prediction server [64].
(DOC)

Table S4 **ADP/O ratios, ACR, RCR, RCR^+^ and Q_10_ analysis (0–15°C) of membrane potentials for complex I and II individually (all values are expressed as means ±SEM).**
(DOC)
