# Supplementary material for: Mitochondrial Function in Antarctic Nototheniids with ND6 Translocation
Source: PLoS One. 2012 Feb 21;7(2):e31860. doi: 10.1371/journal.pone.0031860 (PMC3283701; doi:10.1371/journal.pone.0031860)
Supplement: Table S1 — ND6, ND2 and COI amino acid sequences compared in this study. The table reports: species names, general description of habitat, protein (aa) sequence GenBank accession number for each protein and reference. (DOC) [file pone.0031860.s001.doc]

| **Species name** | **Habitat** | **ND6 aa_GenBank acc. No.** | **References** | **ND2 aa_GenBank acc. No.** | **References** | **COI aa_GenBank acc. No.** | **References** |
| --- | --- | --- | --- | --- | --- | --- | --- |
| ***Notothenia coriiceps***  (Perciformes, Nototheniidae) | Polar (Antarctic, marine) | ACZ62678 | Zhuang and Cheng 2010 | AAP82777 | Cheng et al. 2003 | YP_004581486 | unpublished |
| ***Notothenia rossii***  (Percifomes, Nototheniidae) | Polar (Antarctic, marine) | ACZ62682 | Zhuang and Cheng 2010 | AAP82781 | Cheng et al. 2003 | ABY56208^a^ | unpublished |
| ***Pachycara brachycephalum***  (Perciformes, Zoarcidae) | Deep water (circum-Antarctic, marine) | ###### | Lucassen et al. personal communication | ######b | Lucassen et al. personal communication | ADW41000 | Dettai et al. 2011 |
| ***Eleginops maclovinus***  (Perciformes, Eleginopidae) | Temperate (Southeast Pacific and Southwest Atlantic, marine) | ABG79682 | Papetti et al. 2007 | ABG79672 | Papetti et al. 2007 | ABG79673 | Papetti et al. 2007 |
| ***Boreogadus saida***  (Gadiformes, Gadidae) | Polar (Panarctic, marine) | YP_001600216 | Breines et al. 2008 | YP_001600206 | Breines et al. 2008 | YP_001600207 | Breines et al. 2008 |
| ***Arctogadus glacialis***  (Gadiformes, Gadidae) | Deep water (Arctic and Northeast Atlantic, marine) | CAP46982 | Breines et al. 2008 | CAP46972 | Breines et al. 2008 | CAP46973 | Breines et al. 2008 |
| ***Gadus morhua***  (Gadiformes, Gadidae) | Temperate (Northwest to Northeast Atlantic, marine) | CAM31967 | Ursvik et al. 2007 | CAM31957 | Ursvik et al. 2007 | CAM31958 | Ursvik et al. 2007 |
| ***Chlorurus sordidus***  (Perciformes, Scaridae) | Tropical (Indo-Pacific coral reefs, marine) | BAD52429 | Mabuchi et al. 2004 | BAD52419 | BAD52419 | BAD52420 | BAD52419 |

**a.** 217 amino acids available only for this species

**b.** 306 amino acids available only for this species
