# Supplementary material for: Mitochondrial Function in Antarctic Nototheniids with ND6 Translocation
Source: PLoS One. 2012 Feb 21;7(2):e31860. doi: 10.1371/journal.pone.0031860 (PMC3283701; doi:10.1371/journal.pone.0031860)
Supplement: Table S3 — Amino acid composition and instability index of the COI protein. The table reports for each species the amino acid composition of COI protein and the instability index computed using the Expasy's ProtParam (http://us.expasy.org/tools/protparam.html) prediction server [64]. (DOC) [file pone.0031860.s003.doc]

|  | **Instability index** | **Ala** | **Arg** | **Asn** | **Asp** | **Cys** | **Gln** | **Glu** | **Gly** | **His** | **Ile** | **Leu** | **Lys** | **Met** | **Phe** | **Pro** | **Ser** | **Thr** | **Trp** | **Tyr** | **Val** |
| --- | --- | --- | --- | --- | --- | --- | --- | --- | --- | --- | --- | --- | --- | --- | --- | --- | --- | --- | --- | --- | --- |
| ***Notothenia coriiceps*** | 28.23 | 9.1 | 1.6 | 2.9 | 2.7 | 0.2 | 1.6 | 2.1 | 8.7 | 3.9 | 8.1 | 11.6 | 1.4 | 4.8 | 8.1 | 5.4 | 5.6 | 7.2 | 3.3 | 3.7 | 7.9 |
| ***Notothenia rossii^a^*** | 22.64 | 10.1 | 1.4 | 4.1 | 3.2 | 0.0 | 2.3 | 0.9 | 9.7 | 1.8 | 8.8 | 16.1 | 0.5 | 5.1 | 6.0 | 6.9 | 6.0 | 6.5 | 2.3 | 2.3 | 6.0 |
| ***Pachycara brachycephalum^a^*** | 24.75 | 10.1 | 1.4 | 4.1 | 3.2 | 0.0 | 2.3 | 0.9 | 9.7 | 1.8 | 7.8 | 16.6 | 0.5 | 5.1 | 6.0 | 6.9 | 6.5 | 6.0 | 2.3 | 2.3 | 6.5 |
| ***Eleginops maclovinus*** | 24.87 | 8.9 | 1.7 | 2.7 | 2.7 | 0.2 | 1.6 | 1.9 | 9.1 | 4.1 | 8.3 | 12.0 | 1.6 | 4.7 | 8.3 | 5.4 | 5.6 | 7.2 | 3.3 | 3.5 | 7.2 |
| ***Boreogadus saida*** | 24.61 | 9.3 | 1.7 | 2.7 | 2.9 | 0.2 | 1.6 | 2.1 | 8.9 | 3.7 | 7.8 | 12.0 | 1.6 | 5.6 | 8.1 | 5.4 | 5.2 | 7.0 | 3.3 | 3.5 | 7.4 |
| ***Arctogadus glacialis*** | 24.61 | 9.3 | 1.7 | 2.7 | 2.9 | 0.2 | 1.6 | 2.1 | 8.9 | 3.7 | 7.8 | 12.0 | 1.6 | 5.6 | 8.1 | 5.4 | 5.2 | 7.0 | 3.3 | 3.5 | 7.4 |
| ***Gadus morhua*** | 24.61 | 9.3 | 1.7 | 2.7 | 2.9 | 0.2 | 1.6 | 2.1 | 8.9 | 3.7 | 7.8 | 12.0 | 1.6 | 5.6 | 8.1 | 5.4 | 5.2 | 7.0 | 3.3 | 3.5 | 7.4 |
| ***Chlorurus sordidus*** | 25.60 | 8.9 | 1.6 | 2.9 | 2.7 | 0.2 | 1.6 | 2.1 | 8.9 | 3.7 | 8.3 | 11.8 | 1.6 | 4.8 | 8.1 | 5.6 | 5.4 | 7.4 | 3.3 | 3.5 | 7.6 |

*protein classified as unstable (threshold: 40)

**a.** 217 amino acids available only for this species
