# Supplementary material for: Mitochondrial Function in Antarctic Nototheniids with ND6 Translocation
Source: PLoS One. 2012 Feb 21;7(2):e31860. doi: 10.1371/journal.pone.0031860 (PMC3283701; doi:10.1371/journal.pone.0031860)
Supplement: Table S4 — ADP/O ratios, ACR, RCR, RCR+ and Q10 analysis (0–15°C) of membrane potentials for complex I and II individually (all values are expressed as means ±SEM). (DOC) [file pone.0031860.s004.doc]

|  | **ADP/O** | **ACR** | **RCR** | **RCR^+^** | **Q_10_ mV/O_2_**  **(membr. potential)** | **Q_10_ stII** | **1/Q_10_** |
| --- | --- | --- | --- | --- | --- | --- | --- |
| ***N. coriiceps* complex I** | 2.65±0.13 | 2.29±0.17 | 2.29±0.29 | 5.57±0.33 | 0.72±0.21 | 2.95±0.75 | 0.46±0.12 |
| ***N. coriiceps* complex II** | 2.24±0.13 | 2.74±0.30 | 3.00±0.20 | 5.49±1.20 | 0.32±0.04 | 5.44±0.72 | 0.20±0.03 |
| ***N. rossii* complex I** | 2.11±0.21 | 1.60±0.11 | 1.79±0.18 | 4.77±0.59 | 0.36±0.04 | 3.07±0.42 | 0.36±0.05 |
| ***N. rossii* complex II** | 1.83±0.21 | 1.95±0.25 | 2.38±0.33 | 4.88±0.46 | 0.41±0.04 | 2.66±0.46 | 0.42±0.05 |
